# Effects of Fortetropin on the Rate of Muscle Protein Synthesis in Older Men and Women: A Randomized, Double-Blinded, Placebo-Controlled Study

**DOI:** 10.1093/gerona/glaa162

**Published:** 2020-06-29

**Authors:** William Evans, Mahalakshmi Shankaran, Edna Nyangau, Tyler Field, Hussein Mohammed, Robert Wolfe, Scott Schutzler, Marc Hellerstein

**Affiliations:** 1 Department of Nutritional Sciences and Toxicology, University of California Berkeley; 2 Division of Geriatrics, Duke University School of Medicine, Durham, North Carolina; 3 Departement of Geriatrics, University of Arkansas for Medical Sciences, Little Rock

**Keywords:** Aging muscle, Muscle protein synthesis, Sarcopenia

## Abstract

**Background:**

Fortetropin is a proteo-lipid complex made from fertilized egg yolk and, in young men, has been shown to increase lean body mass.

**Methods:**

The purpose of this study was to examine the effects of 21 days of Fortetropin supplementation on the fractional synthetic rate (FSR) of muscle protein in 10 healthy, older men and 10 women (66.4 ± 4.5 y). We used ^2^H_2_O labeling to measure FSR of multiple muscle protein ontologies. D_3_-creatine dilution was used to determine muscle mass at baseline. Subjects ingested 70% ^2^H_2_O for 21 day and saliva samples were collected to determine body ^2^H_2_O enrichment. A microbiopsy was obtained from the m. vastus lateralis on Day 21. Subjects were randomly assigned to Fortetropin (19.8 g/d) or placebo (cheese powder, 19.8 g/d).

**Results:**

Restricting kinetic data to proteins with ≥2 peptides measured in at least 4 subjects per group resulted in 117 proteins meeting these criteria. The mean FSR for a majority of proteins in several muscle gene ontologies was higher in the Fortetropin group compared to placebo (32/38 myofibril proteins, 33/44 sarcoplasmic proteins, and 12/17 mitochondrial proteins) and this proportion was significantly different between groups using a binomial test and were independent of sex or baseline muscle mass.

**Conclusions:**

The overall magnitude of the difference in muscle protein FSR of Fortetropin from placebo was 18%, with multiple gene ontologies affected. While these results should be confirmed in larger cohorts, they suggest that Fortetropin supplementation is effective for promoting muscle protein synthesis in older people.

Sarcopenia has been defined as the age-related loss of skeletal muscle mass (1). In older people, low muscle mass is strongly associated with reduced functional capacity and an increased risk of disability (2). Recent studies demonstrate that muscle mass has a powerful effect on risk of disability, fall, and poor functional capacity (2). While basal rates of muscle protein synthesis may not change with age (3), sarcopenia is, at least in part, a result of a reduced rate of protein synthesis after a protein containing meal, referred to as anabolic resistance (4). This age-related reduction in postprandial muscle protein synthesis rate has a number of causes, including decreases in testosterone and growth hormone levels, insulin resistance, reduced levels of physical activity, and more. However, skeletal muscle is also a remarkably plastic tissue with multiple pathways that can stimulate hypertrophy or cause atrophy.

Myostatin is a negative regulator of muscle growth (also referred to as growth differentiation factor-8 or GDF-8), is a member of the transforming growth factor-β superfamily of growth and differentiation factors, and has become an important target for pharmaceutical companies as a way to increase muscle protein synthesis and growth. Anti-myostatin drugs increase muscle size and strength in preclinical studies. Clinical studies with anti-myostatin therapy or activin II receptor blockade in older people have shown significant increases in lean body mass and small increases in functional capacity (5,6). Fortetropin is a proteo-lipid complex made from fertilized egg yolk and Sharp and colleagues (7) demonstrated that Fortetropin provided as a supplement lowered circulating myostatin levels in rodents and in young men in combination with resistance exercise also lowered myostatin, increased lean body mass, and increased mTOR signaling compared to placebo. The functional significance and interpretation of circulating myostatin levels is uncertain, however.

The rate of synthesis of multiple muscle proteins in vivo can be measured using tandem mass spectrometric analysis of labeling patterns after ingestion of relatively small amounts of ^2^H_2_O to enrich total body water. Deuterium is incorporated through intermediary metabolic pathways into free amino acids which then enter newly synthesized proteins. In this way, a proteomic approach to changes in the fractional synthetic rate (FSR) of proteins is possible. We previously demonstrated in rats (8) that a selective androgen receptor modulator (a muscle anabolic drug) had a potent dose–responsive effects on the FSR of multiple muscle proteins, particularly in the myofibrillar and glycolytic gene ontologies, and that the short-term increases in FSR were strongly related to longer-term muscle hypertrophy. We have also shown in human subjects, using the heavy water labeling approach with tandem mass spectrometric analyses, that resistance exercise training in obese older men increased FSR of multiple muscle proteins across all ontologies (9) and that a sprint exercise training regimen in young men and women increased FSR of muscle proteins, mostly in the glycolytic and structural protein ontologies (10).

We hypothesized here that compared to controls, daily consumption of Fortetropin supplements would increase the FSR of skeletal muscle proteins.

## Methods

This study was approved by the institutional review board of University of Arkansas for Medical Sciences. A total of 20 healthy men and women were recruited, provided informed consent, and were enrolled in the study. Subjects (mean age 66.4 ± 4.5 years) were randomly assigned to a treatment (FO) or control group (CO). Those in the treatment group consumed Fortetropin (19.8 g/d) and the placebo control group consumed cheese powder 19.8 g/d that was matched for macronutrient and energy to Fortetropin (egg yolk) for 21 days. Central randomization was accomplished by the diet staff which prepared the Fortetropin and placebo supplements and placed them in similar looking containers with a specific study number. The study investigators, staff, or research volunteers were not made aware of the study group assignments. No dietary controls were required and subjects were asked to maintain their normal activity patterns during the 21-day treatment period but food intake was not controlled or assessed. Three days before the initiation of FO or CO, each participant ingested a 30 mg capsule of D_3_-creatine for the measurement of muscle mass. After 3 days, subjects reported to the laboratory and produced a fasting urine sample for later analysis of D_3_-creatinine enrichment, creatine, and creatinine concentrations (11), which were used for the estimation of baseline muscle mass. On Days 1 and 21, a blood sample was collected for determination of circulating myostatin levels by ELISA (GDF-8/Myostatin Quantikine ELISA Kit, R&D Systems).

On the first four days of treatment, subjects ingested three 50 mL quantities of 70% ^2^H_2_O to rapidly (bolus) increase body water enrichment. Thereafter (Days 5–21), subjects consumed 50 mL of 70% ^2^H_2_O to maintain a constant body water enrichment. Saliva samples were collected on Days 7, 14, and 21 for determination of ^2^H_2_O enrichment (12). A microbiopsy (13) (approximately 10 mg) was collected and immediately frozen for determination of muscle proteome-wide FSR (14).

Muscle proteome dynamics was measured using previously described methods (8,14,15). Briefly, muscle biopsy tissue was suspended in 0.08% SDS at a 10:1 volume:weight ratio, and vortexed at low speed overnight (16 h) to extract cellular proteins. The SDS-soluble proteins were precipitated by overnight incubation at −20°C in ethanol (5:1 ethanol:extraction buffer) followed by centrifugation at 16 000*g* for 45 min. Pelleted proteins were rinsed twice with 90% ethanol, allowed to air dry, and resuspended in 8 M urea prior to trypsin digestion. Up to 80 µg of SDS-soluble protein sample was denatured using Protease-Max surfactant (0.1%; Promega, Madison, WI) and 4 M urea in 25 mM ammonium bicarbonate (pH 8). Proteins were reduced with TCEP (5 mM) for 20 min at room temperature with vortexing and then incubated with iodoacetamide (10 mM) in the dark for 20 min to chemically modify reduced cysteines. Proteins were then digested with trypsin (Promega) at 37°C overnight using a 1:25 trypsin:protein mass ratio. The following day, formic acid was added to a total concentration of 5%, and samples were centrifuged at 14 000*g* for 30 min. The supernatant was transferred to a fresh tube, desalted with a C18 spec tip (Varian, Palo Alto, CA), dried via vacuum centrifugation, and resuspended in 0.1% formic acid/3% acetonitrile prior to LC/MS analysis.

Trypsin-digested peptides were analyzed on a 6550 QTOF (quadrupole time-of-flight) mass spectrometer with a 1260 Chip Cube nano ESI source (Agilent Technologies, Santa Clara, CA). Each sample was analyzed once for protein/peptide identification in data-dependent MS/MS mode and once for peptide isotope analysis in MS mode. Acquired MS/MS spectra were extracted and searched using Spectrum Mill Proteomics Workbench software (Agilent Technologies) and mouse protein database (UniProt.org). Search results were validated with a global false discovery rate of 1%. A filtered list of peptides was collapsed into a nonredundant peptide formula database containing peptide elemental composition, mass, and retention time. This was used to extract mass isotope abundances (M0–M3) of each peptide from MS-only acquisition files with Mass Hunter Qualitative Analysis software (Agilent Technologies). An in-house software was used to calculate peptide elemental composition and curve fit parameters for predicting peptide isotope enrichment (EM0) based on precursor body water enrichment (p) and the number (n) of amino acid C-H positions per peptide actively incorporating hydrogen (H) and deuterium (D) from body water.

Subsequent data handling was performed using python-based scripts, with input of average body water enrichment for each participant, to yield fractional synthesis data at the protein level. FSR data were filtered to only include protein measurements with ≥2 peptide isotope measurements per protein measured in at least four subjects per group. Additional details of the FSR calculations and data filtering criteria were as described in detail previously (10,14).

The sample analysis was performed on de-identified samples. Statistical analyses were performed for the different groups of proteins by *t*-test with Benjamini–Hochberg correction for multiple comparisons, as well as by Binomial test on magnitude of change for gene ontological groups of myofibril, cytoplasmic, and mitochondrial proteins.

## Results

**Figure 1.**
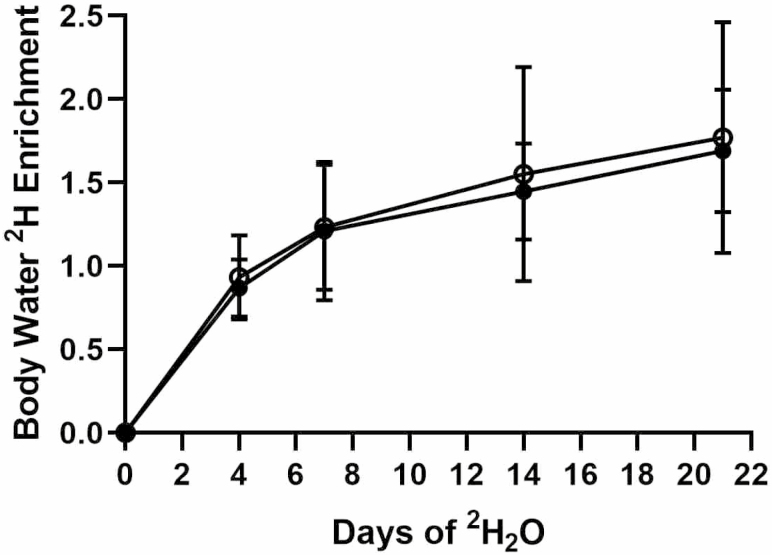
Total body ^2^H_2_O enrichment during 21 days of daily intake of 70% ^2^H_2_O. No differences between groups was observed. Fortetropin: closed circles, Control: open circles.

All 20 subjects enrolled completed all aspects of this study. Enrichment of ^2^H_2_O increased over 3 weeks to approximately 1.5% of total body water and was not different between the two study groups (Figure 1). No differences in baseline muscle mass between FO (30.81 ± 8.46 kg) and CO (26.06 ± 9.30 kg) were observed, data represent average ± *SD*.

### Muscle protein FSR

MS/MS analysis identified 210 muscle proteins with ≥2 peptides/protein. Using analytic criteria of ≥2 peptides per protein measured in at least 4 subjects per group, the kinetic data comprise 109 muscle proteins. Fractional synthesis for each participant was calculated using the average body water used as the precursor enrichment and FSR (% per week) was calculated as −ln(1−*f*)/*t*, where (*f*) is fractional synthesis and (*t*) the duration in weeks of label.

**Figure 2.**
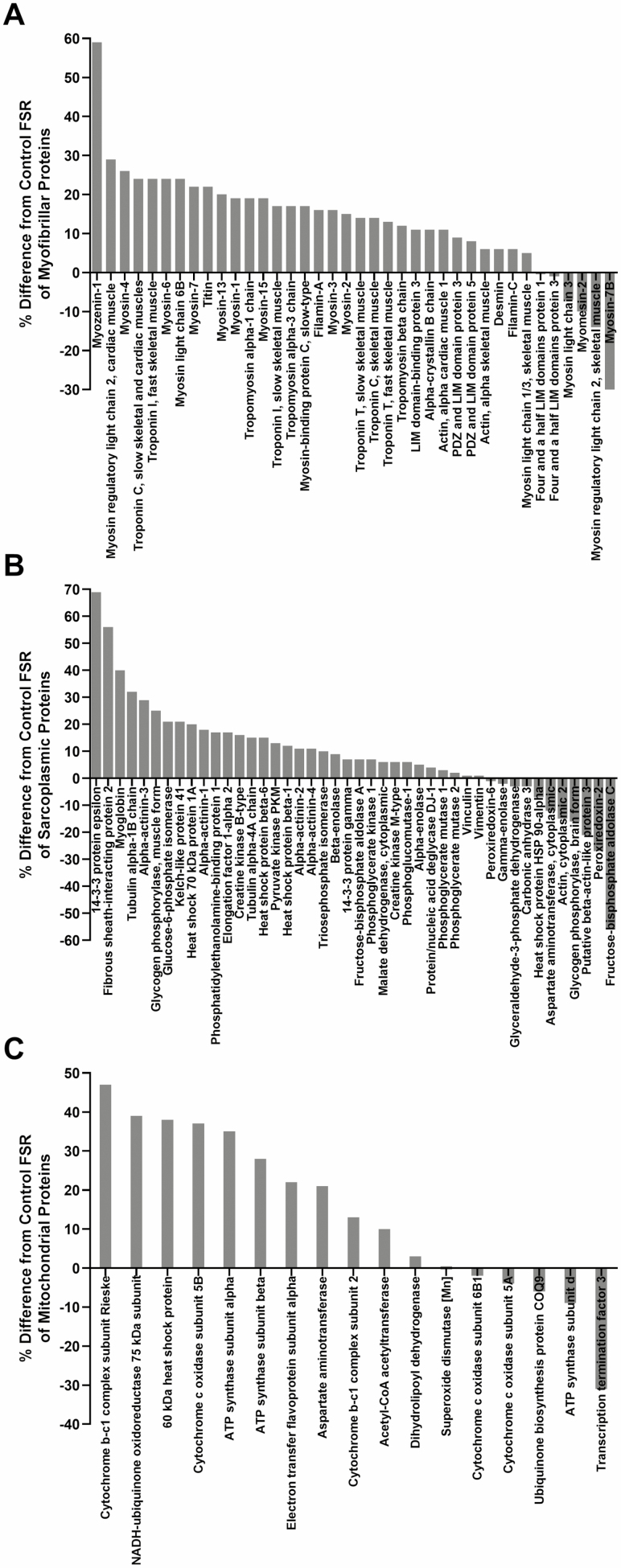
Relative differences (%) in the fractional rate of synthesis for individual muscle proteins by genetic ontology between Fortetropin versus control groups (A) myofibrillar, (B) sarcoplasmic, (C) mitochondrial.

Fractional synthetic rate was measured for all samples, however the number of subjects used for each protein measurement varies based on the analytical criteria of requiring two or more peptides per protein. Each protein must be measured in four or more subjects per group. The FSR of myofibril, sarcoplasmic, and mitochondrial proteins measured in FO and CO groups is given in Table 1 (sample size for each protein is shown). Although there were no significant differences using a Bonferroni correction among individual proteins, an overall 18 ± 13% greater FSR was observed in the FO group for the average of 109 muscle proteins. The average FSR for a majority of proteins in several gene ontologies was higher in the FO group (32/38 myofibril proteins, 33/44 sarcoplasmic proteins, and 12/17 mitochondrial proteins as shown in Figure 2A, B, and C, respectively) and these ontology proportions were each different from control (*p* < .05 by the binomial test).

**Table 1.** Fractional Synthetic Rate (FSR) of Individual Muscle Proteins (±*SD*) by Gene Ontology for Fortetropin and Control Groups

Muscle Protein	Fortetropin FSR	Control FSR	% Difference
Myofibrillar			
Myozenin-1	23.56 ± 5.35 (6)	14.85 ± 5.22 (7)	59
Myosin regulatory light chain 2, ventricular/cardiac muscle isoform	23.51 ± 6.39 (10)	18.20 ± 5.34 (10)	29
Myosin-4	9.82 ± 3.51 (10)	7.79 ± 3.29 (10)	26
Troponin C, slow skeletal and cardiac muscles	19.37 ± 4.67 (10)	15.65 ± 4.53 (10)	24
Troponin I, fast skeletal muscle	16.00 ± 6.15 (8)	12.94 ± 6.94 (8)	24
Myosin-6	7.72 ± 3.89 (9)	6.24 ± 2.42 (9)	24
Myosin light chain 6B	9.77 ± 2.59 (10)	7.91 ± 2.06 (10)	24
Myosin-7	8.19 ± 2.30 (10)	6.73 ± 2.46 (10)	22
Titin	18.17 ± 5.56 (10)	14.96 ± 5.05 (10)	22
Myosin-13	7.65 ± 2.02 (10)	6.36 ± 2.21 (10)	20
Myosin-1	22.76 ± 5.62 (10)	19.06 ± 5.26 (10)	19
Tropomyosin α-1 chain	9.07 ± 2.20 (10)	7.60 ± 2.22 (10)	19
Myosin-15	11.66 ± 2.69 (10)	9.82 ± 3.20 (9)	19
Troponin I, slow skeletal muscle	8.84 ± 2.34 (10)	7.53 ± 2.26 (10)	17
Tropomyosin α-3 chain	6.19 ± 3.47 (9)	5.30 ± 1.93 (10)	17
Myosin-binding protein C, slow-type	9.73 ± 2.14 (10)	8.34 ± 2.74 (10)	17
Filamin-A	11.23 ± 1.73 (8)	9.65 ± 1.33 (6)	16
Myosin-3	9.55 ± 1.63 (10)	8.23 ± 2.23 (10)	16
Myosin-2	9.49 ± 1.82 (10)	8.25 ± 2.11 (10)	15
Troponin T, slow skeletal muscle	24.15 ± 7.06 (7)	21.12 ± 14.86 (9)	14
Troponin C, skeletal muscle	13.73 ± 6.34 (10)	12.05 ± 9.02 (10)	14
Troponin T, fast skeletal muscle	12.42 ± 3.48 (10)	11.01 ± 4.75 (10)	13
Tropomyosin-β chain	4.28 ± 1.07 (10)	3.82 ± 1.74 (10)	12
LIM domain-binding protein 3	8.67 ± 1.45 (8)	7.80 ± 1.40 (6)	11
α-Crystallin B chain	13.78 ± 5.09 (10)	12.39 ± 4.96 (10)	11
Actin, α-cardiac muscle 1	24.38 ± 5.28 (10)	21.94 ± 640 (9)	11
PDZ and LIM domain protein 3	7.05 ± 2.43 (9)	6.49 ± 2.33 (10)	9
PDZ and LIM domain protein 5	2.68 ± 1.47 (10)	2.47 ± 1.28 (10)	8
Actin, α-skeletal muscle	4.43 ± 1.11 (10)	4.16 ± 1.52 (10)	6
Desmin	32.83 ± 13.77 (10)	30.87 ± 17.79 (10)	6
Filamin-C	18.55 ± 7.65 (9)	17.50 ± 8.55 (10)	6
Myosin light chain 1/3, skeletal muscle isoform	3.99 ± 0.75 (10)	3.79 ± 0.84 (10)	5
Four and a half LIM domains protein 1	25.30 ± 9.19 (6)	25.39 ± 11.09 (10)	0
Four and a half LIM domains protein 3	26.19 ± 0.128 (4)	26.52 ± 2.29 (5)	−1
Myosin light chain 3	8.47 ± 2.22 (8)	9.08 ± 3.66 (8)	−7
Myomesin-2	19.33 ± 3.85 (8)	21.47 ± 7.03 (7)	−10
Myosin regulatory light chain 2, skeletal muscle isoform	4.92 ± 1.52 (10)	5.72 ± 3.81 (10)	−14
Myosin-7B	5.28 ± 2.71 (5)	7.52 ± 3.91 (5)	−30
Mean magnitude of increase			17
Binomial test two-tailed *p*-value			*p* < .0001
Sarcoplasmic			
14-3-3 Protein epsilon	17.06 ± 7.25 (5)	10.12 ± 5.34 (6)	69
Fibrous sheath-interacting protein 2	14.32 ± 3.72 (5)	9.18 ± 4.42 (5)	56
Myoglobin	5.42 ± 2.52 (10)	3.87 ± 1.96 (10)	40
Tubulin α-1B chain	25.69 ± 4.45 (4)	19.44 ± 7.44 (4)	32
α-Actinin-3	5.72 ± 1.49 (10)	4.44 ± 1.62 (10)	29
Glycogen phosphorylase, muscle form	13.96 ± 3.37 (9)	11.21 ± 3.94 (10)	25
Glucose-6-phosphate isomerase	6.37 ± 2.32 (4)	5.25 ± 2.60 (6)	21
Kelch-like protein 41	20.09 ± 4.27 (4)	16.60 ± 3.46 (5)	21
Heat shock 70 kDa protein 1A	18.68 ± 5.52 (4)	15.63 ± 2.17 (6)	20
α-Actinin-1	6.21 ± 1.60 (8)	5.25 ± 1.63 (6)	18
Phosphatidylethanolamine-binding protein 1	11.76 ± 4.59 (6)	10.04 ± 2.55 (4)	17
Elongation factor 1-α 2	18.51 ± 2.31 (6)	15.81 ± 4.00 (6)	17
Creatine kinase B-type	5.80 ± 1.88 (10)	4.99 ± 2.95 (10)	16
Tubulin alpha-4A chain	24.91 ± 7.07 (4)	21.62 ± 2.98 (5)	15
Heat shock protein β-6	24.65 ± 13.41 (9)	21.40 ± 8.32 (10)	15
Pyruvate kinase PKM	7.02 ± 2.17 (7)	6.24 ± 2.10 (8)	13
Heat shock protein β-1	18.53 ± 4.46 (9)	16.56 ± 5.28 (9)	12
α-Actinin-2	6.29 ± 1.54 (10)	5.66 ± 1.81 (10)	11
α-Actinin-4	5.69 ± 1.57 (8)	5.13 ± 1.70 (6)	11
Triosephosphate isomerase	7.58 ± 1.61 (9)	6.92 ± 2.75 (10)	10
β-Enolase	8.63 ± 3.77 (10)	7.88 ± 3.36 (10)	9
14-3-3 Protein gamma	17.12 ± 6.74 (4)	15.97 ± 3.78 (5)	7
Fructose-bisphosphate aldolase A	10.01 ± 2.67 (10)	9.35 ± 3.60 (10)	7
Phosphoglycerate kinase 1	3.89 ± 2.32 (6)	3.63 ± 2.14 (7)	7
Malate dehydrogenase, cytoplasmic	6.94 ± 2.92 (8)	6.53 ± 3.60 (10)	6
Creatine kinase M-type	6.98 ± 1.73 (10)	6.57 ± 2.73 (10)	6
Phosphoglucomutase-1	8.42 ± 1.08 (8)	7.97 ± 2.62 (9)	6
α-Enolase	7.08 ± 3.37 (10)	6.76 ± 3.41 (10)	5
Protein/nucleic acid deglycase DJ-1	8.07 ± 1.68 (5)	7.72 ± 0.91 (5)	4
Phosphoglycerate mutase 1	4.93 ± 1.21 (9)	4.78 ± 1.79 (10)	3
Phosphoglycerate mutase 2	4.00 ± 1.80 (10)	3.93 ± 1.73 (10)	2
Vinculin	16.17 ± 3.26 (8)	15.93 ± 2.34 (6)	1
Vimentin	23.34 ± 6.66 (7)	23.19 ± 8.61 (8)	1
Peroxiredoxin-6	9.49 ± 1.97 (7)	9.58 ± 5.05 (8)	−1
γ-Enolase	7.19 ± 2.50 (10)	7.33 ± 4.23 (10)	−2
Glyceraldehyde-3-phosphate dehydrogenase	8.34 ± 1.84 (10)	8.57 ± 7.51 (9)	−3
Carbonic anhydrase 3	4.42 ± 1.73 (10)	4.58 ± 2.01 (10)	−3
Heat shock protein HSP 90-α	10.34 ± 2.73 (8)	11.08 ± 3.48 (4)	−7
Aspartate aminotransferase, cytoplasmic	10.43 ± 3.39 (8)	11.86 ± 2.37 (10)	−12
Actin, cytoplasmic 2	4.48 ± 2.55 (8)	5.25 ± 3.07 (6)	−15
Glycogen phosphorylase, brain form	14.39 ± 4.60 (7)	17.14 ± 6.71 (6)	−16
Putative β-actin-like protein 3	3.30 ± 1.47 (8)	4.20 ± 0.54 (6)	−21
Peroxiredoxin-2	6.91 ± 4.17 (5)	9.42 ± 5.41 (8)	−27
Fructose-bisphosphate aldolase C	8.40 ± 1.06 (6)	19.16 ± 17.84 (8)	−56
Mean magnitude of increase			16
Binomial test two-tailed *p*-value			*p* < .0005
Mitochondrial			
Cytochrome b-c1 complex subunit Rieske, mitochondrial	14.72 ± 5.43 (6)	10.02 ± 3.05 (4)	47
NADH-ubiquinone oxidoreductase 75 kDa subunit, mitochondrial	79.78 ± 24.34 (4)	57.49 ± 36.90 (5)	39
60 kDa heat shock protein, mitochondrial	8.47 ± 3.77 (8)	6.11 ± 1.41 (4)	38
Cytochrome c oxidase subunit 5B, mitochondrial	11.93 ± 4.80 (5)	8.68 ± 5.10 (5)	37
ATP synthase subunit α, mitochondrial	12.70 ± 3.80 (5)	9.38 ± 1.87 (7)	35
ATP synthase subunit β, mitochondrial	10.57 ± 3.12 (10)	8.27 ± 2.59 (10)	28
Electron transfer flavoprotein subunit α, mitochondrial	11.77± 5.65 (8)	9.65 ± 4.06 (8)	22
Aspartate aminotransferase, mitochondrial	7.26 ± 2.50 (6)	5.99 ± 1.38 (5)	21
Cytochrome b-c1 complex subunit 2, mitochondrial	10.87 ± 3.93 (4)	9.65 ± 1.87 (5)	13
Acetyl-CoA acetyltransferase, mitochondrial	8.65 ± 2.61 (10)	7.85 ± 1.01 (10)	10
Dihydrolipoyl dehydrogenase, mitochondrial	10.66 ± 3.71 (8)	10.31 ± 3.87 (9)	3
Superoxide dismutase [Mn], mitochondrial	11.89 ± 3.85 (8)	11.87 ± 4.58 (8)	0.1
Cytochrome c oxidase subunit 6B1	9.76 ± 3.72 (8)	9.93 ± 5.99 (7)	−2
Cytochrome c oxidase subunit 5A, mitochondrial	10.70 ± 2.39 (8)	11.12 ± 3.27 (9)	−4
Ubiquinone biosynthesis protein COQ9, mitochondrial	6.56 ± 3.16 (4)	7.02 ± 3.98 (5)	−6
ATP synthase subunit d, mitochondrial	4.48 ± 1.59 (4)	4.95 ± 1.89 (7)	−9
Transcription termination factor 3, mitochondrial	5.62 ± 1.03 (6)	8.13 ± 5.03 (5)	−31
Mean magnitude of increase			25
Binomial test two-tailed *p*-value			*p* < .05

% Differences and average % differences by ontology are provided.

**Figure 3.**
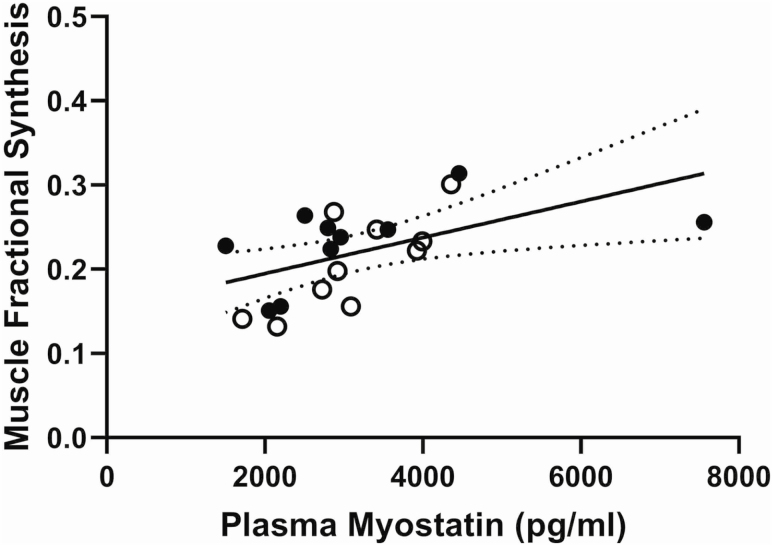
The relationship between circulating myostatin levels on Day 21 versus average muscle protein FSR. Fortetropin: closed circles, Control: open circles (*r* = 0.5327, *p* < .05).

No change in circulating myostatin during the 21-day treatment period and no differences in myostatin concentrations between the two groups were seen. FO: 3.09 ± 1.29 ng/mL, CO: 2.96 ± 0.69 ng/mL at baseline; FO: 3.24 ± 1.72 ng/mL, CO: 3.12 ± 0.83 ng/mL after 21 days of treatment (data represent mean ± SD). A significant positive correlation (*r* = 0.5327, *p* < .05) between circulating myostatin concentrations and the average muscle protein fractional synthesis on Day 21 was seen (Figure 3) with a significant correlation of similar strength for Day 1 myostatin levels (*r* = 0.4688, *p* < .05).

## Discussion

The major finding of this double-blinded study is that, compared to placebo (cheese powder), daily use of the nutritional supplement Fortetropin resulted in a significant increase in the rate of synthesis muscle proteins as a group in healthy older men and women. The increased muscle protein FSR was observed in the major ontologies of muscle proteins—sarcoplasmic, myofibrillar, and mitochondrial proteins. Although significant increases in FSR were not observed for individual muscle proteins, the majority of proteins in all three ontologies had higher mean FSRs in the Fortetropin group (32/38 myofibril proteins, 33/44 sarcoplasmic proteins, and 12/17 mitochondrial proteins), each of which were statistically significant by the binomial test.

This study is consistent with a previous study demonstrating an effect of Fortetropin consumption to increase lean body mass in young subjects. While the mechanism of this increase in muscle protein FSR was not examined, Sharp and colleagues (7) showed that in combination with exercise in rats and resistance trained young men, Fortetropin increased mTOR expression. Fortetropin also resulted in a decrease in circulating myostatin levels. Although we did not observe any effect on circulating myostatin levels, Fortetropin has been shown to decrease circulating myostatin levels (7,16) in healthy subjects. The consensus of published data suggests an age associated decrease in circulating myostatin levels (17,18), although the role of myostatin in aging has not been established and the functional interpretation of circulating myostatin remains uncertain. We found here that in a group of healthy older subjects, circulating myostatin levels were positively associated with muscle protein FSR. A positive correlation is, however, contrary to the hypothesized relationship (myostatin is expected to inhibit muscle protein synthesis). This relationship was similar and significant whether Day 1 or Day 21 levels of myostatin were used. Myostatin has a role in increasing muscle ubiquitination and rate of muscle protein breakdown (19). The relationship between muscle protein synthesis rates and circulating myostatin may be a result of higher breakdown of muscle proteins and the resultant increase in free amino acids which have a stimulatory effect on muscle protein FSR. Our data do not support a direct role of circulating myostatin in the effects of Fortetropin to increase FSR of muscle proteins.

^2^H_2_O labeling for determination of protein FSR provides an integrated measurement during the period of labeling that includes both fed and fasted state assessments. In the present study, ^2^H_2_O was consumed by subjects for a 21-day period and a broad effect of Fortetropin was observed across the muscle proteome, with a significant increase in the FSR of myofibrillar, mitochondrial, and sarcoplasmic proteins. Using this heavy water labeling-tandem mass spectrometric method in rats, we previously showed that a selective androgen receptor modulator resulted in a substantial and dose–responsive increase in muscle protein FSR, more so in glycolytic and myofibrillar than in mitochondrial protein ontologies, after 10 days that was strongly associated with increased muscle mass after 28 days of use (8). These findings demonstrated that the muscle protein synthetic anabolic response is predictive of the magnitude of muscle hypertrophy. Using heavy water labeling, we have previously demonstrated that resistance exercise increases skeletal muscle protein FSR by about 25% in older men (9), comparable to the 18% increase seen in the present study.

In our study, subjects ingested ^2^H_2_O during the entire treatment period of 21-days and as a result, the FSR values represent the average rate of synthesis during that period of time, including both postprandial and postabsorptive conditions. As a result, it is not possible to know if the anabolic effect of Fortetropin occurred in combination with meals or in the postabsorptive condition. Anabolic resistance describes the reduced stimulation of muscle protein synthesis in response to specific amount of protein or essential amino acids in older subjects compared to healthy young people (4) and has been suggested as important in the etiology of sarcopenia. Indeed, levels of protein intake are associated with lean body mass (20) and healthy older people have a higher requirement for dietary protein (21) than is currently recommended for the general population. The subjects in our study were healthy and diet was not controlled during the supplementation period. The macronutrient content of Fortetropin and placebo was the same and the subjects and investigators were blinded as to which study group each participant was part of. The higher rates of muscle protein synthesis suggest that Fortetropin may work to overcome anabolic resistance of aging. Stimulation of muscle protein FSR will result in improvements in muscle mass. Recent studies now demonstrate that muscle mass is strongly associated with health-related outcomes, risk of disability, and mortality in older men (2,22).

In conclusion, in healthy older men and women the daily use of Fortetropin resulted in higher synthesis rates of muscle proteins of multiple ontologies compared to a control group. The effects were independent of sex or initial level of muscle mass. The ability to measure integrated synthesis rates of large numbers of proteins over several weeks’ time by the dynamic proteomics method, using tandem mass spectrometric analyses with long-term heavy water labeling, provided here a sensitive approach for detecting subtle differences in global protein synthesis rates.

These data suggest Fortetropin has an anabolic effect on muscle protein synthesis in older people and warrants testing as a therapy for sarcopenia. The long-term effects of Fortetropin on muscle mass and function in older people is unknown and should be explored.
